# Toxigenic *Vibrio cholerae* O1 in vegetables and fish raised in wastewater irrigated fields and stabilization ponds during a non-cholera outbreak period in Morogoro, Tanzania: an environmental health study

**DOI:** 10.1186/s13104-016-2283-0

**Published:** 2016-10-18

**Authors:** Yaovi M. G. Hounmanou, Robinson H. Mdegela, Tamègnon V. Dougnon, Ofred J. Mhongole, Edward S. Mayila, Joseph Malakalinga, George Makingi, Anders Dalsgaard

**Affiliations:** 1 Department of Veterinary Medicine and Public Health, Sokoine University of Agriculture, Morogoro, Tanzania; 2 Research Laboratory in Applied Biology, Polytechnic School of Abomey-Calavi, University of Abomey-Calavi, Abomey-Calavi, Benin; 3 National Health Laboratory Quality Assurance and Training Centre (NHLQATC), Dar Es Salaam, Tanzania; 4 Faculty of Health and Medical Sciences (UC-HEALTH), University of Copenhagen, Copenhagen, Denmark

**Keywords:** *Vibrio cholerae* O1, Wastewater, Fish, Vegetables, Antibiotic susceptibility

## Abstract

**Background:**

Cholera, one of the world’s deadliest infectious diseases, remains rampant and frequent in Tanzania and thus hinders existing control measures. The present study was undertaken to evaluate the occurrence of toxigenic *Vibrio cholerae* O1 in wastewater, fish and vegetables during a non-outbreak period in Morogoro, Tanzania.

**Methods:**

From October 2014 to February 2015, 60 wastewater samples, 60 fish samples from sewage stabilization ponds and 60 wastewater irrigated vegetable samples were collected. Samples were cultured for identification of *V. cholerae* using conventional bacteriological methods. Isolates were confirmed as *V. cholerae* by detection of the outer membrane protein gene (*omp*W) using polymerase chain reaction (PCR). Isolates were further tested for antibiotic susceptibility and presence of virulence genes including, cholera enterotoxin gene (*ctx*), the toxin co-regulated pilus gene (*tcpA*) and the haemolysin gene (*hly*A).

**Results:**

The prevalence of *V. cholerae* in wastewater, vegetables and fish was 36.7, 21.7 and 23.3 %, respectively. Two isolates from fish gills were *V. cholerae* O1 and tested positive for c*tx* and *tcp*A. One of these contained in addition the *hly*A gene while five isolates from fish intestines tested positive for *tcp*A. All *V. cholerae* isolates were resistant to ampicillin, amoxicillin and some to tetracycline, but sensitive to gentamicin, chloramphenicol, and ciprofloxacin.

**Conclusions:**

Our results show that toxigenic and drug-resistant *V. cholerae* O1 species are present and persist in aquatic environments during a non-cholera outbreak period. This is of public health importance and shows that such environments may be important as reservoirs and in the transmission of *V. cholerae* O1.

## Background

Climate change is one of the leading causes of water scarcity which is a serious problem in the world and in developing countries in particular. For populations experiencing water scarcity, a main source of water is reclaimed wastewater which is used for a large range of activities including fish culture [1]. Fish culture and vegetables irrigation using wastewater is practiced by populations living in water scarce areas in Tanzania [1]. This situation is due to the depletion of the natural fish stock and harvests from freshwater and ocean fisheries [2] and the increased demand of vegetables alongside high water scarcity. However, fish and vegetables grown in such unhygienic conditions may represent food safety hazards to consumers, i.e. as documented by the isolation of a number of pathogens including *Vibrio cholerae*, the causative agent of cholera [3, 4]. In cholera endemic areas, the disease often has a seasonal pattern [5, 6] and cholera remains highly frequent in Tanzania due to multiple reasons that need to be addressed [7, 8]. Since the seventh cholera pandemic reached the country in 1974, the disease has been reported almost every year in Tanzania regardless of seasons [7, 8]. It is therefore likely that neglected reservoirs of *V. cholerae* exist in Tanzania. Inadequate information on possible sources of toxigenic strains of *V. cholerae* complicates cholera prediction, prevention and control and contributes to frequent and unexpected outbreaks of cholera in Tanzania. Therefore, there is a need to assess to what extent toxigenic strains of *V. cholerae* occur in the aquatic environment during non-cholera outbreak periods. Widespread use of antibiotics as prophylaxis during cholera outbreaks worldwide has contributed to the development of multidrug resistant strains of *V. cholerae* [9]. Information and surveillance of the antibiotic susceptibility of the organism not only in clinical, but also in environmental isolates in endemic regions is thus needed. The present study aimed to determine the occurrence of toxigenic *V cholerae* and their antibiotic susceptibility in isolates obtained from fish and vegetables grown in wastewater during a non-cholera outbreak period in Morogoro, Tanzania.

## Methods

### Study sites

The study was carried out at Mafisa and Mzumbe wastewater treatment units and at a vegetable production site called Funga–Funga near the Morogoro River in urban and peri-urban Morogoro, Tanzania. The Mafisa wastewater treatment unit receives municipal wastewater mainly from households, commercial areas and hospitals around Morogoro. Treated wastewater from Mafisa is used to irrigate a large area of rice fields. Funga–Funga is the main and largest vegetable cultivation site located alongside Morogoro River with fields being irrigated with water from the river. Mzumbe wastewater treatment unit is located at the University of Mzumbe and receives wastewater from housing facilities, cafeterias and the hospital. The treated wastewater is discharged through an informal network of earth canals and used to irrigate a variety of vegetables.

### Collection of samples

**Fig. 1:**
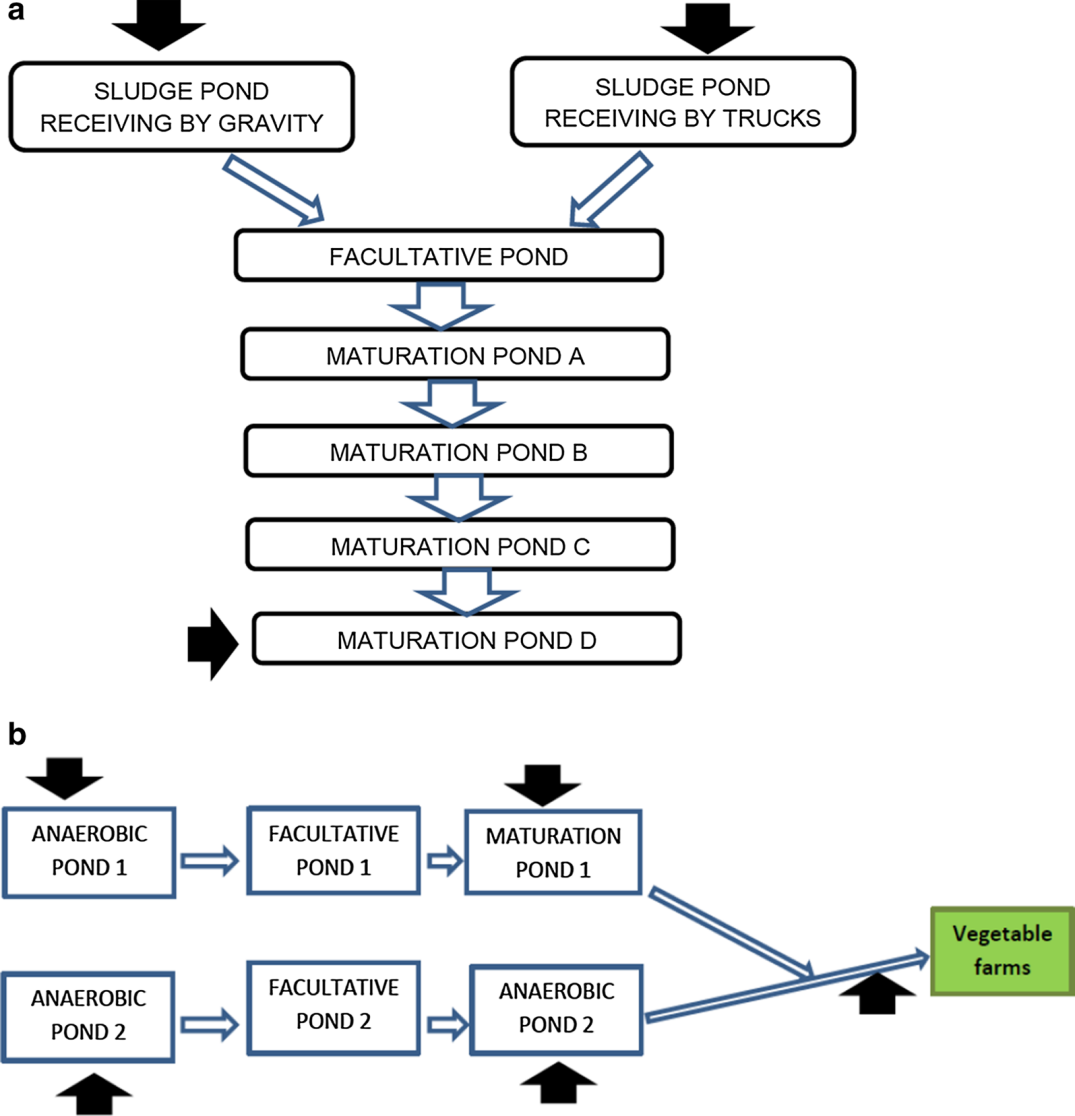
Water sampling points at Mafisa (**a**) and Mzumbe (**b**) wastewater treatment units. *Black arrows* indicate sampling points

A cross-sectional study was conducted from October 2014 to February 2015 including samples of wastewater, Chinese cabbage (*Brassica rapa*), Nile tilapia (*Oreochromis niloticus)*, and African sharp tooth catfish (*Clarias gariepinus*). Water samples were collected during five samplings at two weeks interval. Fifteen water samples were collected from different points of Mafisa and 20 water samples were collected along Morogoro River and 25 samples from Mzumbe. From the ponds, water samples were collected from the inlets and the outlets (Fig. 1). Volumes of 100 ml were collected in sterile bottles according to WHO guidelines for microbiological drinking water analysis [10]. A total of ten Chinese cabbage heads were collected from each field irrigating with treated effluent from the Mzumbe and Funga–Funga production sites and placed in sterile plastic bags during each of three monthly samplings with cabbage heads obtained from different furrows during each sampling. Thirty tilapia and 30 African sharp tooth catfish of at least 300-grs size were collected by cast net from Mzumbe stabilisation ponds early in the morning and individually placed in sterile plastic bags. Samples were placed in an insulated box with cooling elements and transported to the laboratory at the Sokoine University of Agriculture in Morogoro where the microbiological analysis was initiated within 2 h of sampling.

### Isolation of *Vibrio cholerae*

In the laboratory, 25 ml of each well mixed water sample was added to 225 ml of Alkaline Peptone Water (APW; (Sigma, Steinheim, Germany)) for enrichment. Approximately, 25 g of cabbage leaves were cut off and added to 225 ml of APW for enrichment. For fish samples, subsamples were individually taken from gills and intestines (10 g of each) and added to 90 ml of APW for enrichment before culture. APW samples were enriched for 18 h at 37 °C then subcultured onto thiosulfate citrate bile sucrose (TCBS) agar (ACCUMIX, Verna, India). Following 24 h incubation at 37 °C, yellow colonies (sucrose fermenting, 2–3 mm diameter) suspected as *Vibrio cholerae* were purified on trypticase soy agar (Merck, Darmstadt, Germany) for 24 h at 37 °C. Purified colonies were characterized by Gram staining (Gram-negative comma shaped rods), positive oxidase reaction (colour change to blue dark within 5 s) and reaction in triple sugar iron agar (yellow slants with no gas formation) [11]. Presumptive *V. cholerae* isolates were confirmed by sero-agglutination test with the polyvalent *V. cholerae* O1 serum (Bio-Rad, Marnes-la-conquette, France).

### Molecular characterization of *Vibrio cholerae*

DNA was extracted from colonies picked from TSA by the boiling lysis method. Following removal of cell debris by centrifugation at 12,000 rpm for 3 min, the supernatant containing the template DNA was stored at −20 °C for PCR [12]. Isolates were confirmed as *V. cholerae* by PCR for the outer membrane protein encoding gene (*Omp*W) [13, 14]. PCR was also done to detect the *ctx*, *tcp*A and *hly*A genes which are all important virulence factors in *V. cholerae* using primers as listed in Table 1.The *ctx* gene is the main virulence factor of *V. cholerae* and encodes the production of cholera toxin which causes the severe diarrhoea seen in cholera patients. *tcp*A intervenes in fimbriae synthesis enabling *V. cholerae* to adhere to the host’s intestinal epithelium whereas the *hly*A gene is associated with blood cell lysis in the infected host. Electrophoresis of amplicons was done in a 1.5 % agarose gel (Advance, Tokyo, Japan) at 100 V for 1 h. The gel was visualized under UV light using Gel Doc EZ Imager apparatus (Bio Rad, California, USA). Double distilled DNase free water was used as negative control and *V. cholerae* O139 NCTC 12945 (ATCC 51394) as a positive control.

**Table 1:** Primers sequences used for the PCR

Targeted genes	Primer Sequences (5′–3′)	Size (bp)	Source
*Ctx* Cholera toxin gene	F-CAGTCAGGTGGTCTTATGCCAAGAGG R-CCCACTAAGTGGGCACTTCTCAAACT	167	[27]
*OmpW* Outer membrane protein	F-CACCAAGAAGGTGACTTTATTGTG R-GAACTTATAACCACCCGCG	588	[3]
*TcpA* Toxin coregulated pilus	F-CAC GAT AAG AAA ACC GGT CAA GAG R-CGA AAG CAC CTT CTT TCA CGT TG	453	[26]
*hlyA* Haemolysin	F-GGC AAA CAG CGA AAC AAA TAC C R-CTC AGC GGG CTA ATA CGG TTT A	727	[6]

### Antimicrobial susceptibility testing

All confirmed *V. cholerae* isolates were subjected to antimicrobial susceptibility testing on Muller Hinton agar plates (OXOID, Basingstoke, Hampshire, England) using the Kirby-Bauer disc diffusion method to the following antibiotics: tetracycline (20 µg), gentamicin (10 µg), ciprofloxacin (5 µg), chloramphenicol (30 µg), ampicillin (10 µg) and amoxicillin (10 µg) (OXOID). Measured inhibition zone diameters were interpreted according to CLSI guidelines [15].

### Data analysis

Proportions of positive *Vibrio cholerae* samples at different sites and different sample types were calculated then compared by Chi square and Fisher exact tests based on the total sizes using EPI-INFO 7 statistical software. Statistical significance was defined at a probability of p = 0.05.

## Results

### Detection of *Vibrio cholerae* in water, vegetables and fish

Out of the 60 water samples, 22 (36.7 %) were positive for *V. cholerae* as shown by a 588 bp sized PCR amplicon of the *Omp*W gene; however none of the isolates did agglutinate the *V. cholerae* O1 antiserum. Water samples collected from the Mafisa unit contained the highest prevalence of *V. cholerae* non-O1 (46.7 %, n = 15), followed by Morogoro River (35.0 %, n = 20) and the Mzumbe unit (32.0 %, n = 25). *V. cholerae* non-O1 was significantly (p < 0.05) more often found in outlet as compared to inlet water at both treatment units.

*Vibrio cholerae* was isolated from 13/60 (21.7 %) Chinese cabbage samples collected from the Mzumbe and Funga–Funga vegetables production sites; however, none of these isolates were *V. cholerae* O1. Cabbage from Funga–Funga site contained a higher *V. cholerae* non-O1 prevalence (36.7 %, n = 30) than did cabbage from Mzumbe production site (6.7 %, n = 30) (p < 0.05).

A total of 15 (25.0 %, n = 60) intestinal samples from all the sampled fish contained *V. cholerae* non-O1 and 13 (21.7, n = 60 %) gill samples contained *V. cholerae* non-O1. Two (7.1 %) of the gill isolates were confirmed as *V. cholerae* O1 in the slide agglutination test. Actually, *V. cholerae* was not found in any catfish samples but only from tilapia.

### Characterization of *Vibrio cholerae* O1 and non-O1

**Fig. 2:**
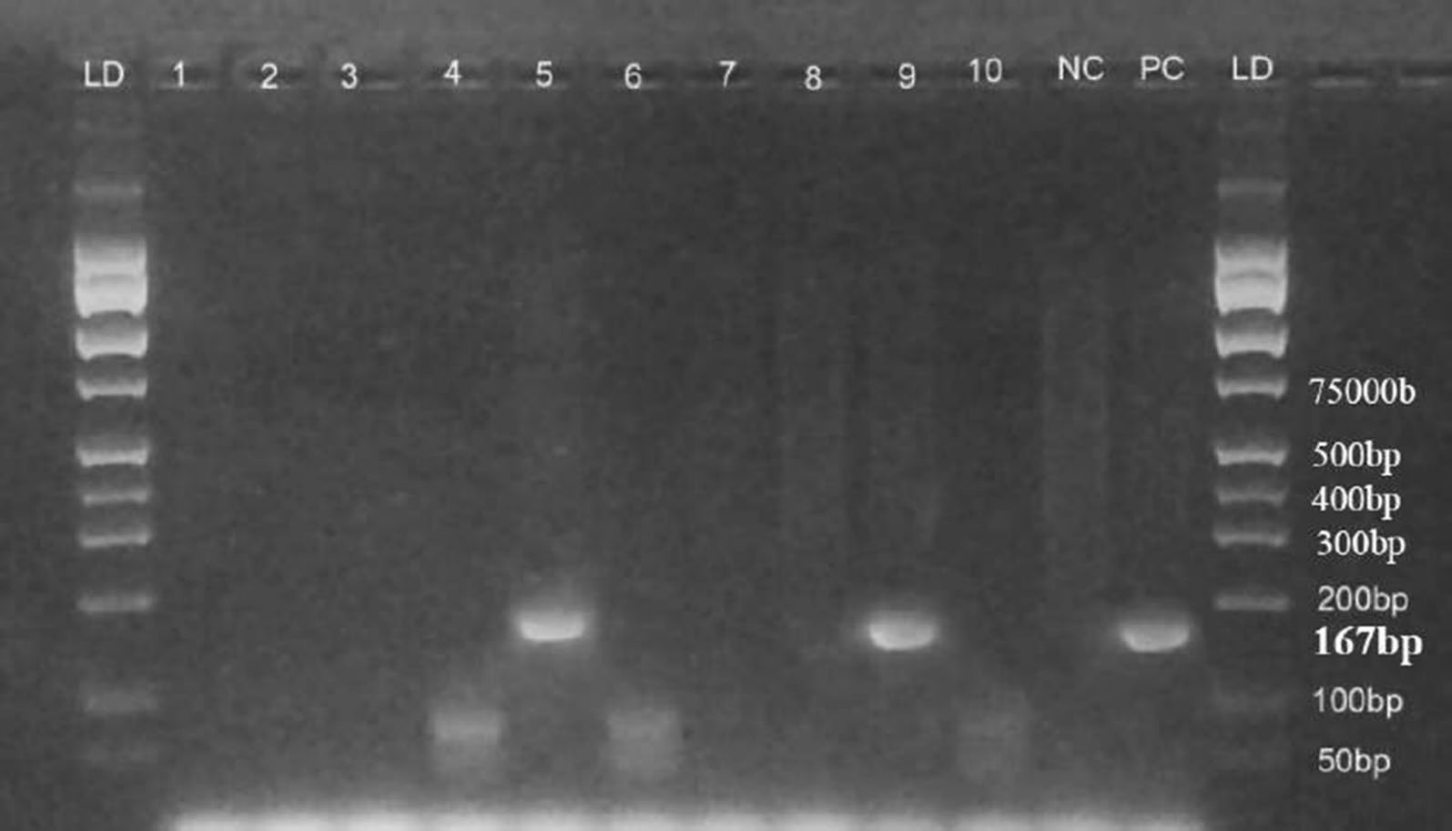
Cholera toxin gene (*ctx*) detected in fish isolates using PCR at 167 bp. *LD* DNA ladder*; lane* 1 to 10 are *V. cholerae* DNA samples*; NC* negative control (DNA free water); *PC* positive control (VC O139, ATCC 51394)

**Fig. 3:**
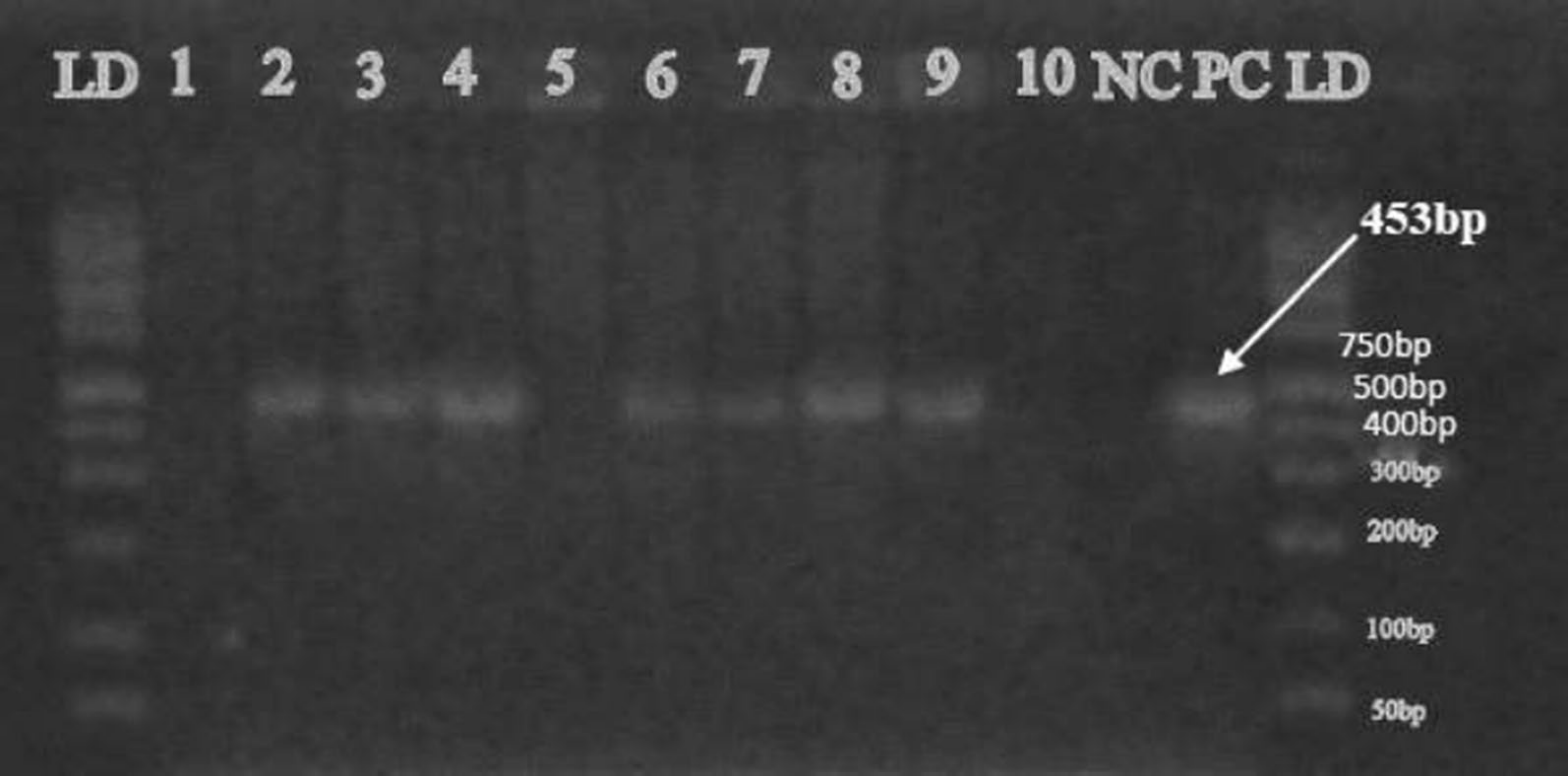
Cholera toxin co-regulated pilus gene (*tcpA*) detected in fish isolates using PCR at 453 bp. *LD* DNA ladder*; Lane* 1 to 10 are *V. cholerae* DNA samples*; NC* negative control; PC positive control

**Fig. 4:**
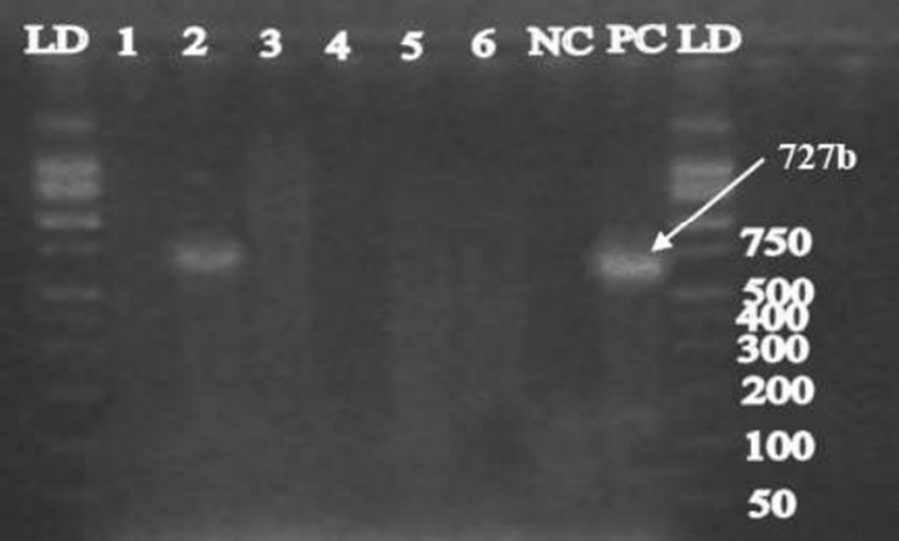
Cholera haemolysin gene (*hly*A) detected in fish isolates using PCR at 727 bp. *LD* DNA ladder, *lane* 1 to 6 are *V. cholerae* DNA samples, *NC* negative control, *PC* positive control

PCR confirmed that the cholera toxin gene (*ctx*) was present in the two *V. cholerae* O1 isolates and the positive control strain as shown by a 167 bp sized amplicon (Fig. 2). The toxin co-regulated pilus subunit A gene (*tcp*-A) was amplified (453-bp, Fig. 3) in seven *V. cholerae* isolates from tilapia including the two *V. cholerae* O1 isolates. Only one of the *V. cholerae* O1 isolates contained the *hlyA* gene (727 bp-sized amplicon, Fig. 4). The two *V. cholerae* O1 isolates, as well as the non-O1 isolates were resistant to ampicillin, amoxicillin and tetracycline, but sensitive to gentamicin, chloramphenicol, and ciprofloxacin.

## Discussion

The isolation of *ctx*-positive *V. cholerae* O1 in a non-cholera outbreak period from fish samples in Tanzania is of food safety and public health concern [8–16].The presence of the toxin co-regulated pilus (*tcp*-A) is important as this gene encodes for fimbriae synthesis which allows the bacteria to adhere to the host’s intestinal epithelium [17]. The haemolysin gene (*hly*A) present in these isolates enables blood cell lysis leading to anaemia in the infected individuals [6]. As the first to ascertain the occurrence of *V*. *cholerae* O1 in fish from a non-outbreak period in Tanzania, this study demonstrates the wide diversity of reservoirs of toxigenic *V. cholerae* O1. This observation is supported by the results of Nkoko et al. [18], who reported various environmental and aquatic reservoirs of *V. cholerae* including blue-green algae and chironomid eggs. Several other studies have illustrated the ability of *V. cholerae* to associate with a number of zooplankton, phytoplankton, blue-green algae (cyanobacteria) that prolong its survival [19, 20]. The isolation of *V. cholerae* O1 from fish samples is of food safety and health concern mainly due to risks for cross- contamination and potential for multiplication in different types of foods. Senderovich et al. [21] confirmed different fish species including tilapia (*Sarotherodon galilaeus*) from aquatic environments as reservoirs of *V. cholerae*. Furthermore, Onyuka et al. [22] isolated *V. cholerae* O1 from fish samples in Kenya during a non-outbreak period and concluded that in cholera endemic areas these microorganisms exist in biofilm-like aggregates in which the cells are in conditional viable state. Similar results were also reported by Sathiyamurthy et al. [3] and Mrityunjoy et al. [4]. Physico-chemical conditions mainly pH and salinity influences the survival of *V. cholerae* O1 [23]. Therefore, the physico-chemical characteristics and conditions in fish gills (the identified reservoir of this study) may favour persistence of such toxigenic organisms in fish. Moreover, Evans et al. [24] reported that in osmoregulation and iron balance in fish, high concentrated chloride solution is secreted by fish gills whereby the mechanisms of NaCl secretion by the gill epithelium are greater than the NaCl uptake from the environment. This suggests that the persistence of toxigenic *V. cholerae* O1 isolates in gills may be associated with NaCl content in this type of tissue. Also, Faruque et al. [19] reported that in natural ecological settings, unidentified environmental factors induce lysogenic phage CTXФ in toxigenic *V. cholerae*, resulting in the release of extracellular CTXФ particles into the aquatic environment. Therefore, the cell-free phage particles may be associated with the emergence of novel toxigenic strains of *V. cholerae* through interactions with non-toxigenic strains. It is uncertain to what extend lysogenic phages like CTXФ play a role in the emergence of toxigenic *V. cholerae* O1 and associated cholera outbreaks in Tanzania where they occur every year in many regions of the country [16]. The frequent cholera outbreaks experienced in Tanzania in recent years may be associated with the ecology and potential aquatic reservoirs of *V. cholerae*, i.e. fish and aquatic organisms. Also, the neighbouring countries of Tanzania are often experiencing cholera outbreaks, e.g. associated with large displacements of people due to social unrest, resulting in discharge and introduction of *V. cholerae* O1 into aquatic environments like the African Great Lakes to which access is shared by Tanzania. Since cholera is a hygiene related disease, most cholera control policies are directed towards household hygiene and sanitation with less attention paid to potential reservoirs and transmission routes of *V. cholerae* O1. An analysis of cholera outbreaks in countries around the African Great Lakes revealed a strong association of cholera with access to and location close to the Lakes [18].

The frequent finding of non-O1 *V. cholerae* in the sewage system may be ecologically normal but the presence of these organisms on irrigated vegetables could represent food safety and human health hazards. Sathiyamurthy et al. [3] demonstrated the clinical and epidemiological importance of non-O1 *V. cholerae* as causes of diarrhoea and gastroenteritis in humans. For instance, non-O1 and non-O139 strains of *V. cholerae* have been associated with occasional diarrhoea outbreaks resembling cholera [25]. Therefore, appropriate measures should be taken to curb the public health threats of diarrhoea associated with consumption of wastewater irrigated vegetable in Tanzania [1]. The prevalence of *V. cholerae* non-O1 in the effluents water of both studied sewage treatment units was higher than those of the inlets. These findings suggest that there is growth and proliferation of the bacteria throughout the system. According to Faruque et al. [19], *V. cholerae* are autochthonous of aquatic environments and can therefore be recovered at any point in a sewage plant independently on those isolated at the inlet. Also, *V. cholerae* may survive better in the latter parts of the treatment system, i.e. in the facultative and the maturation ponds where water is cleaner and contains less competitive microorganisms. Besides, studies have illustrated the ability of *V. cholerae* to associate with a variety of zooplankton, phytoplankton, blue-green algae (cyanobacteria) that prolong its survival and facilitate multiplication [19, 20]. Such organisms are often seen in high concentrations in the maturation ponds. The recovery of high concentrations of *V. cholerae* in effluent water indicates that wastewater stabilization ponds are unable to effectively remove these pathogens and could be considered as reservoirs of toxigenic *V. cholerae O1* during non-cholera outbreak period.

Although rehydration plays a pivotal role in reducing mortality during cholera epidemics, antibiotics have been used to reduce the shedding of the organism (thereby reducing spread of the disease), treating severe illness (by reducing volume of diarrhoea), and also to reduce duration of disease and hospitalisation [9]. However, the resistance of microorganisms including *V. cholerae* to antibiotics has become a serious health challenge worldwide. Our *V. cholerae* O1 isolates were resistant to ampicillin, amoxicillin and tetracycline; a resistance pattern that has also been reported elsewhere for *V. cholerae* O1 [23–28]. Such similarity could be due to the misuse of the same antibiotics all over the world because in this era of antibiotics resistance, effective antibiotics are communicated and prescribed worldwide leading to simultaneous occurrence of resistance around the world. The resistance to tetracycline is particularly worrisome as tetracycline has been shown to be effective treatment for cholera and quite superior to other antimicrobials in reducing cholera morbidity. It should be noted that our *V. cholerae* O1 strains were sensitive to gentamicin, chloramphenicol and ciprofloxacin with the latter being a recommended alternative drug for treatment of cholera cases. The misuse and release of antibiotics in the sewage could be one of the reasons of occurrence of antibiotics resistant organisms in these environmental isolates.

## Conclusion

Different serotypes of *V. cholerae* were isolated from wastewater and its irrigated vegetables and fish in Morogoro, Tanzania from October 2014 to February 2015 during a non-outbreak period. Two isolates were confirmed *V. cholerae* O1. Three main virulence genes were detected in these isolates notably the cholera toxin gene, the leading cause of the disease and others virulent genes mainly the toxin co-regulated pilus and the haemolysin gene which play important roles in the pathogenesis of the bacteria. The *V. cholerae* O1 isolates demonstrated resistance to ampicillin, amoxicillin and tetracycline, but sensitivity to gentamicin, chloramphenicol, and ciprofloxacin. Thus, toxigenic *V. cholerae* O1 were present and seem to persist in environmental samples during a non-cholera outbreak period. Such an environmental reservoir is of food safety and public health concern and shows that environmental reservoirs of *V. cholerae* O1 should be studied and monitored as part of cholera prevention and control programmes.
